# How to improve dealing with desire to die in hospice volunteers and informal caregivers

**DOI:** 10.1017/S1478951525000239

**Published:** 2025-03-14

**Authors:** Kathleen Boström, Thomas Dojan, Axel Doll, Thomas Montag, Raymond Voltz, Kerstin Kremeike

**Affiliations:** 1 Department of Palliative Medicine, Faculty of Medicine and University Hospital, University of Cologne, Cologne, NRW, Germany; 2 Center for Integrated Oncology Aachen Bonn Cologne Duesseldorf (CIO ABCD), Faculty of Medicine and University Hospital, University of Cologne, Cologne, NRW, Germany; 3 Center for Health Services Research, Faculty of Medicine and University Hospital, University of Cologne, Cologne, NRW, Germany

**Keywords:** Desire to die, informal caregivers, hospice volunteers, palliative and hospice care, online trainings

## Abstract

**Objectives:**

Up to 40% of severely ill patients report at least an occasional desire to die, opening up not only to professionals but also to hospice volunteers and patients’ informal caregivers. Based on an existing, evaluated 2-day desire to die training for professionals, we intend to adapt the training for hospice volunteers and informal caregivers, both face-to-face and online and provide a preliminary evaluation.

**Methods:**

Multi-method approach to (1) assess needs regarding content and form for (online) trainings for hospice volunteers and formats for informal care givers using online focus groups and (additional) individual interviews, (2) adapt existing training materials for both groups accompanied by expert discussion, and (3) pilot and evaluate the (online) trainings and formats through (online) surveys.

**Results:**

In an online focus group with *n* = 4 informal caregivers and *n* = 2 additional online interviews, participants reported wishes for form (e.g. short formats in plain language) and content (e.g. needs in relation to health professional and patient). The *n* = 6 hospice volunteers also wished for form (e.g. plain language) and content (e.g. volunteer role). Results were implemented in (a) a volunteer adaptation of the training, e.g. with target-group-specific case studies and (b) the development of an online format for informal caregivers. For evaluation, we conducted (a) 2 face-to-face trainings for hospice volunteers (*n* = 14 and *n* = 20) and (b) 2 online formats for informal caregivers (*n* = 7 and *n* = 13). Both groups benefited strongly from participation.

**Significance of results:**

Hospice volunteers and informal caregivers deal with patients’ desires to die – often without being adequately prepared. Through (online) trainings and formats, their awareness and self-confidence regarding desire to die can increase. It is therefore of high relevance to meet the demand for easily accessible and target group specific (online) trainings on dealing with desire to die.

## Introduction

When confronted with severe and life-limiting disease, desires to die are a common reaction in patients with 12–45% reporting temporary and 10–18% persistent desires to die (Chochinov et al. 1995; Wilson et al. 2016). We define desire to die along a continuum of increasing suicidal pressure. Forms along this continuum can range from life satiety, tiredness of life to the (hypothetical) wish for hastened death and can manifest (in extreme forms) as suicidality or the wish for assisted suicide (Belar et al. 2021; German Guideline Program in Oncology 2020). Desires to die are prone to change over time in quality and intensity and do not rule out a concurrent will to live (Boström et al. 2024; German Guideline Program in Oncology 2020; Voltz et al. 2010).

Palliative and hospice care providers are often confronted with patients’ desires to die and report support needs in dealing with this sensitive topic (Galushko et al. 2016). Therefore, we developed a 2-day face-to-face training on theoretical knowledge, communicative skills, and self-reflection regarding desire to die (Frerich et al. 2020). Training participants reported a stable increase of self-confidence in dealing with desire to die, e.g. by feeling able to address it proactively – even 1 year later (Boström et al. 2022). To this day, our training is still in high demand which prompted a further development into both an online adaptation and a website (Boström et al. 2025). However, a need for modifications became apparent. Most of the training participants worked in medical, nursing, psychosocial, and pastoral sectors or volunteered in inpatient and outpatient hospice care (Boström et al. 2022). During the multi-professional trainings conducted so far, hospice volunteers offered different perspectives and needs regarding the accompaniment of patients with a desire to die.

Informal caregivers are also affected by patients’ desires to die. As closest to the patient, they are present throughout the whole illness trajectory and on most hours of the day. Therefore, they face a double burden: not only providing a high amount of informal care but also coping with their own distress and anticipatory grief (Goldberg et al. 2021). Communication about patients’ desires to die is often hampered by a mutual fear of burdening the other party (Gudat et al. 2019). Moreover, desires to die are often hard to deal with for informal caregivers; they may struggle with the feeling of not having done enough for their close one or they feel guilty due to fostering those wishes themselves (Balasubramanian et al. 2024; Harstäde et al. 2013). However, there are – to our knowledge – no established and evaluated information or exchange formats for this target group in Germany yet.

Attending a 2-day face-to-face training on dealing with desire to die is not feasible for all (informal) care providers, e.g. due to time constraints involved in care work or travel costs. Since the onset of the COVID-19 pandemic, online provision of medical education in form of webinars has shown to be a potential alternative with a wide outreach (Quraishi et al. 2024).

Therefore, we aimed to adapt the existing 2-day face-to-face training (Frerich et al. 2020) according to target-group specific needs for (online) education on desire to die in two forms:

1. (online) modification of our 2-day training adapted to the needs of hospice volunteers
2. (online) information and exchange format for informal caregivers of patients with desire to die

Both formats are to be followed by a preliminary evaluation as proof of feasibility.

## Methods

Based on our original multi-professional 2-day training on dealing with desire to die (Frerich et al. 2020), we aimed to adapt content and form for hospice volunteers and informal caregivers. We therefore followed a threefold procedure:

1. Online focus groups with hospice volunteers and informal caregivers (representatives) to collect data on experiences and needs for content and form of educational formats
2. Target group-specific adaption of the existing training program, accompanied by expert discussion within the project team
3. Piloting and preliminary evaluation of developed (online) trainings

All research was conducted according to the Declaration of Helsinki and received a favorable vote from the ethics committee of the University of Cologne (Nr. 21–1412_1, 04.11.2022).

### Online focus groups with hospice volunteers and informal caregivers

We planned 2 online focus groups with *N* = 5–10 participants each, 1 with hospice volunteers and 1 with informal caregivers dealing with desire to die. For focus group moderation, we created a semi-structured interview guideline (Helfferich 2004) (see Supplement 1). For recruitment, we contacted 7 self-help groups, 1 hospice association, and a known contact person at 1 university hospital by mail which they forwarded to potential participants. All participants received a gift voucher as incentive and gave written informed consent.

We used the platform Zoom to conduct and record the online focus groups (Zoom Video Communications 2011). Two members of the research team (KB, TD) moderated the focus group, with one primarily guiding the group and the other taking notes. Based on these notes, a *short transcript* was created. Verbatim quotes and missing content was added from audio recordings (Schulz et al. 2012). If there were less focus group participants than aimed at, supplementary individual interviews were planned as backup, following the same procedure.

The qualitative data analysis followed the process laid out by Miles et al. (2018): In a first cycle, data were coded using descriptive and sub-coding with subsequent theming of the codes, if adequate. Coded data was then clustered into main- and subthemes. For better visual clarity, we generated tables to display main- and subthemes. Qualitative analysis was supported by the software MAXQDA 2022 (VERBI Software 2021).

### Target-specific adaption of the existing training curriculum

The findings from the online focus groups were discussed within the project team regarding practicability of their implementation. The project team included *N* = 6 research team members and/or course instructors from the existing 2-day training curriculum, representing different areas of expertise (teaching and training, psychology, social sciences, nursing, and palliative care).

### Piloting and preliminary evaluation

We planned to conduct 4 newly developed (online) training formats: Two for hospice volunteers and 2 for informal caregivers. For each target group 1 face-to-face and 1 online format was intended to provide the same content and use comparable didactic methods.

#### Online training for hospice volunteers

For evaluation of our adapted training curriculum, we used a questionnaire on self-confidence in dealing with the desire to die (Frerich et al. 2020). It was to be distributed to participants prior to (t0) and immediately following (t1) the 2 trainings. The self-developed questionnaire comprises 22 items pertaining to knowledge, skills and attitudes regarding desire to die (Frerich et al. 2020). Answers could be given on a 5-point Likert-scale ranging from 1 (“Do not agree”) to 5 (“Fully agree”), with the exception of item 1 using a 7-point Likert-scale. For the online training, the questionnaire was converted into an online survey utilizing the LimeSurvey platform.

#### Online format for informal caregivers

For the evaluation of our online information and exchange format for informal caregivers, we developed and used a short online survey with 4 open questions:

1. Do you think that your participation in this event will help you meet your challenges in dealing with desire to die? Please explain your answer.
2. Are there any aspects of desire to die that have not been covered today that you think should be included? Please explain them.
3. How do you rate the implementation in an online format?
4. What did you particularly like about the event? What can we do better from your point of view? Do you have any further comments?

Participants were to receive a link to the platform LimeSurvey to answer the questions and give basic sociodemographic information.

All answers to open questions from both evaluation of the (online) training for hospice volunteers and the (online format) for informal caregivers were transferred to MAXQDA 22 (VERBI Software 2021) for qualitative data analysis. Statistical data were analyzed descriptively in IBM SPSS Statistics 29 for means, standard deviations, and frequencies (IBM Corp Released 2017).

## Results

There was a wealth of experience and needs from the focus groups to (a) adapt our trainings for hospice volunteers and (b) develop an information and exchange format for informal caregivers. As planned, we conducted 4 trainings for subsequent preliminary evaluation (c) but adapted their format (online vs. face-to-face) according to participant needs. Results will be reported by target group: hospice volunteers first and informal caregivers second.

### Hospice volunteer trainings

#### Online focus group with hospice volunteers

We recruited *n* = 6 hospice volunteers for participation in an online focus group. All participants were female, aging between 50 and 59 (*n* = 4) and 60–69 (*n* = 2). Professional backgrounds included online education (*n* = 3), psychosocial care (*n* = 1), and other (*n* = 1).

Overall, input on needs and expectations of hospice volunteers was categorized into main- and subthemes, presented in Table 1 and explained in more detail below.

**Table 1.** Main themes and subthemes for needs and expectations for education formats aimed at hospice volunteers and informal caregivers as derived from the focus groups

Main themes	Subthemes
**Hospice volunteers**	
**Online trainings**	Requested content
	Requested form
	Suggestions for organization (e.g. single or serial dates)
	Structure of the online training (duration, number of participants, combination of exchange and education)
	Creating an atmosphere of trust
	Inclusion of analog media
	Networking via other channels
**Important aspects for volunteers**	Self-reflection of own attitude
	Knowledge of desire to die
	Own role between informal caregivers and health professionals
	Dealing with patients directly
**Promotion and preparation of online education for volunteers**	Preparatory assignments
	Online methods
	Networking for participants
	Self-exploration
**Informal caregivers**	
**Important aspects for health professionals when dealing with informal caregivers**	Knowing own role (e.g. carer, information provider)
	Education about desire to die
	Offer of support in case of emotional stress
	Care for informal caregivers
**Important aspects for informal caregivers**	Dealing with own experiences and emotions
	Knowledge about desire to die
	Seeking support with health professionals
	Dealing with own desire to die

As *important aspects for volunteers*, they want their special position to be acknowledged in (online) trainings on dealing with desire to die. They characterize their *special role* and self-image as more distant to patients than informal caregivers, but closer than health professionals. They see their mission as *companionship instead of care* and describe their task as follows:

We are the only ones who don’t want anything from the patients, except of doing them good. A nurse has a clear mission [to deliver care work]. We have a mission, too, but with a different focus. (Volunteer, online focus group)

Volunteers benefit from their significantly larger time contingent compared to health professionals. They expressed a need for further training in *knowledge* and dealing appropriately with patients who desire to die specifically from their own perspective. *Requested forms* for to achieve this could be by using case studies written in accessible language (i.e., featuring less biomedical terminology) and focusing on relevant (e.g., psychosocial instead of biomedical) aspects. Participants also report uncertainty regarding their own role as volunteers in case assisted suicide is administered as *further requested content*. For online trainings, volunteers suggest *preparatory assignments* (e.g., introductory reading), theatre pedagogical tools and educational films with simulated patients. They also prompt the incorporation of *analog media* in video formats by making creative use of the camera (e.g. arranging and filming objects in their room).

#### Adaptation of training for hospice volunteers

The results of the online focus group were synthesized and implemented with emphasis on practical feasibility. For the perspective of volunteers, we were able to adapt our current course material according to wishes reported in the focus group, especially our case studies. Language was adapted to include fewer medical terms and more psychosocial information as well as a stronger volunteer perspective. In addition, questions for (self-)reflection were developed for each exercise that explicitly refer to the volunteer role, e.g. in talking about a wish for assisted suicide. Apart from that, many suggestions by volunteers were already reflected in the existing training curriculum (e.g. “self-reflection of own attitude,” “knowledge of desire to die,” “creating an atmosphere of trust,” “dealing with patients directly” and “self-exploration”), therefore strengthening the case for a pragmatic approach.

#### Preliminary evaluation of trainings for hospice volunteers

##### Recruitment and participant characteristics

For the hospice volunteer trainings, regional ambulatory palliative care services were contacted who had previously shown interest in the training on dealing with desire to die. Eventually, implementation differed from the original study design for the evaluation of the hospice volunteer training: due to the strong demand for face-to-face trainings, we dispensed with online conduction, but offered 2 face-to-face trainings in 2 ambulatory hospice services with *N* = 14 participants in June 2023 and *N* = 20 participants in November 2023, respectively. Table 2 shows sociodemographic information of participants of the 2 hospice volunteer trainings.

**Table 2.** Sociodemographic data for all participants of the hospice volunteer trainings and informal caregiver formats on dealing with patients’ desire to die

	Hospice volunteers		Informal caregivers	
	Face-to-face I (*n* = 14)	Face-to-face II (*n* = 20)	Online I (*n* = 7)	Online II (*n* = 13)
*Age*				
≤19−39	/	/	1	/
40−49	1	/	2	2
50−59	4	2	3	5
60−70+	9	17	2	2
Not specified	/	1	/	/
*Gender*				
Female	13	17	6	8
Male	1	3	1	1
*Profession*^a^				
Physician	/	/	/	/
Nursing	3	5	/	/
Psychosocial	3	2	7	/
Volunteer	12	20	/	/
Relative^b^	2	3	7	9
Other	/	/	/	/

a Multiple answers possible.

b All participants who chose the “relative”-option also chose another option.

##### Preliminary evaluation

Hospice volunteers who took part in the face-to-face trainings completed an evaluation questionnaire before and directly after the training (Frerich et al. 2020). Mean values were not normally distributed according to the Shapiro–Wilk test (up to *p* < .001). A Wilcoxon test was therefore carried out to compare mean values. Mean values and standard deviations of all items can be found in Table 3.

**Table 3.** Mean values and standard deviations (in brackets) of all items at t0 (before training) and t1 (after training) for the face-to-face trainings of hospice volunteers

		Face-to-face I (*n* = 14)		Face-to-face II (*n* = 20)	
No.	Item	t0	t1	t0	t1
1	How confident do you feel to discuss a patient’s desire to die?	4.00 (1.13)	5.44* (0.53)	4.41 (1.50)	5.27* (0.96)
2	I would feel discomfort in discussing desire to die with patients.	2.07 (0.67)	1.69* (0.63)	2.05 (1.05)	1.45* (0.69)
3	Desire to die discussions are not possible for me due to lack of time.	1.29 (0.47)	1.23 (0.44)	1.40 (0.75)	1.20* (0.52)
4	I am afraid that a discussion with a patient about his/her desire to die would affect me too deeply.	2.21 .893	1.92 (0.64)	2.15 (1.04)	1.50* (0.76)
5	I am able to address desire to die proactively with a patient.	2.71 (0.91)	2.23 (0.72)	2.45 (1.36)	2.42 (1.22)
6	I know different ways of reacting to patients with a desire to die.	2.57 (0.76)	4.38* (0.51)	2.50 (1.23)	3.68* (0.95)
7	I am able to use different approaches to respond to patients with desires to die.	2.57 (0.76)	4.15* (0.55)	2.15 (0.99)	3.40* (0.88)
8	I know several possible backgrounds to a desire to die.	3.14 (1.03)	4.54* (0.66)	3.53 (1.17)	4.30* (0.73)
9	I know several possible functions of a desire to die.	2.64 (0.84)	4.46* (0.52)	2.60 (1.14)	4.25* (0.72)
10	I am familiar with the current legal situation regarding physician-assisted suicide. allowing people to die and euthanasia.	2.36 (1.08)	4.38* (0.51)	2.90 (1.33)	4.25* (1.02)
11	I am unsure about my duty of care with suicidal patients.	2.93 (1.21)	2.31 (0.85)	3.56 (0.98)	2.47* (0.87)
12	I know the key points of relevant recommendations for dealing with desires to die.	2.50 (0.94)	4.15* (.89)	2.05 (0.86)	3.68* (0.58)
13	I know signs that indicate acute suicidal tendencies in patients.	2.15 (0.89)	3.69* **(.85)**	2.70 (0.92)	3.40* (0.68)
14	I recognize signs of own exhaustion when confronted with desire to die.	3.07 (0.92)	3.62 (0.96)	3.30 (0.92)	3.80* (0.89)
15	I can manage to not exhaust myself when patients express a desire to die.	3.36 (0.84)	3.92* (0.86)	3.00 (0.92)	3.75 (0.97)
16	I am not aware of my own attitude to the subject of desire to die.	3.00 (1.18)	2.00 (1.41)	2.15 (1.42)	1.80* (1.28)
17	I am aware of my fears when dealing with patients with desire to die.	3.31 (0.95)	3.77 (1.01)	3.65 (1.09)	4.10 (0.85)
18	When a patient asks me for help in dying I discuss this desire to die with the patient in detail.	3.43 (0.85)	4.08 (0.76)	3.65 (0.87)	4.35* (1.04)
19	When I am confronted with a desire to die. I feel helpless.	2.21 (0.69)	1.92 (0.86)	2.40 (0.82)	2.00 (1.08)
20	When I am confronted with a desire to die. I want to flee the situation.	1.93 (0.62)	1.6 (0.51)	2.05 (0.94)	2.05 (1.39)
21	I am able to accept patients with their desires to die.	3.71 (1.20)	4.15 (1.07)	3.80 (1.32)	4.30 (1.03)
22	I am able to stay in contact with patients with desires to die.	3.71 (1.14)	4.15 (1.08)	4.10 (1.12)	4.37 (1.01)

* Significant with *p* ≤ 0.05.

Hospice volunteers showed more significant improvements between t0 and t1 based on quantity of items with significant differences between t0 and t1 than multiprofessional palliative care providers did (Boström et al. 2025): 11 of 22 items (items 1 (*p*=.007), 2 (*p* = 027), 5 (*p* = .023), 6 (*p* <. 001), 7 (*p* <.001), 8 (*p* <.001), 10 (*p* <.001), 12 (*p* = .040), 14 (*p*=.004), 15 (*p* < .001), 16 (*p* < .001), 17 (*p* = .012), 18 (*p* = .044)) and 15 of 22 items for the second (items 1 (*p* = .019), 2 (*p* = .007), 3 (*p* = .021), 4 (*p* = . 012), 6 (*p* = .002), 7 (*p* = < .001), 8 (*p* < .001), 9 (*p* < .001), 10 (*p* < .001), 11 (*p* = .028), 12 (*p* = .005), 13(*p* = .029), 14 (*p* = .004), 15 (*p* < .001), 16 (*p* < .001) 18 (*p* = .011), and 21 (*p* = .043). Thereby, training participants reported significant improvements on all 3 levels of knowledge, skills, and attitude in dealing with desire to die in at least half of all items.

### Informal caregiver formats

#### Online focus group with informal caregivers

We recruited *n* = 4 informal caregiver representatives as participants and conducted *n* = 2 supplementary individual interviews with informal caregivers. Participants were mostly female (*n* = 5), aging between 40 and 49 (*n* = 4) 50–59 (*n* = 1) and ≥70 (*n* = 1). For their expert background, multiple answers were possible. As some were representatives of informal caregiver, only *n* = 4 of the participants reported themselves to be informal caregivers. Others reported volunteer work (*n* = 3), work as a physician (*n* = 1) and work in nursing or psychosocial care (*n* = 2).

Findings on informal caregivers could be categorized into main- and subthemes. For a general overview, see Table 1.

Overall, participants recommend focusing on *education about desire to die*, by provision of knowledge and correction of misinformation as well as extra attention to the *concerns and suffering of informal caregivers*. A short length (1–2 h) for up to 5 participants was deemed appropriate.

Informal caregivers’ interaction with health professionals and the relationship with patients that harbor a desire to die were reflected. Focus group participants with a professional background distinguished 2 possible *roles for informal caregivers*: they are either a resource and assist in care or they themselves can need care when regarded as co-suffering. To meet the *emotional needs of informal caregivers* burdened by patient suffering, health professionals should offer support through respect, acceptance, and help in normalizing (potentially intense) emotions of informal caregivers. If informal caregivers themselves develop a desire to die (including suicidality), health professionals should be obliged to act according to suicide prevention. In the case of particularly close relationships between informal caregivers and patients, the question of who actually holds the desire to die may arise: is it the informal caregivers or the dying person? One informal caregiver representative suggests:

..the lives [of patients and informal caregivers] may be so inextricably linked that perhaps a clear-cut difference no longer even exists. (Informal caregiver representative (physician), online focus group)

Participants in the online focus groups also considered topics such as *informal caregivers’ emotions*, e.g. uncertainty, hope, guilt, and acceptance to be important. Uncertainty and guilt can make it difficult to acknowledge the desire to die they might have been confronted with:

At first, you will probably try to offer some sort of consolation or point to other perspectives, [..] and just hope that perhaps [the desire to die] was not meant as seriously. (Informal Caregiver, online focus group)

To foster acceptance, *knowledge about desire to die* is described as beneficial, e.g. being aware of the link between pain and desire to die or the difference between desire to die and suicidality. Informal caregivers who suffer from the desire to die of their accompanied person should request *health professional support* for themselves.

#### Development of an information and exchange format for informal caregivers

For informal caregivers of patients who desire to die, a 2-h online format was developed, consisting of a 30-min scientific input on desire to die conveyed in lay-terms and a moderated 90-min experience exchange. Input on desire to die was comprised of empirical knowledge on background, meaning and functions, suggestions for communication about it as well as interventions to deal with desire to die with their respective legal framework. The format was led in tandem by a scientist/psychologist and a hospice coordinator/grief counselor.

#### Preliminary evaluation of (online) formats for informal caregivers

##### Recruitment and participant characteristics

For informal caregivers, recruitment for the first online information and exchange formats was carried out by (1) contacting known a regional informal caregivers association and (2) inviting a total of 16 supra-regional self-help groups and associations of informal caregivers of people with various life-limiting illnesses. The initiative of a participant contributed significantly to the recruitment for a second online format, who recruited for a second appointment using social media via her connection to a self-help organization.

Through these recruitment measures, the format was held twice in the evening hours via Zoom (Zoom Video Communications 2011), to allow working informal caregivers to participate. In March 2023, *n* = 7 and in August 2023, *n* = 13 participants joined the online format. Due to the high demand for the digital format, there was a waiting list of *N* = 14 interested parties after the first event, who were also contacted again for the second event. Interestingly, we recorded an opposite trend in the demand for online formats than we did with hospice volunteers. Counter the original study design, both implemented formats were online. Socio-demographic information of participants can be found in Table 2.

##### Evaluation of information and exchange format for informal caregivers

Open-ended questions from the online survey revealed an overall positive reception of the online format. Participating informal caregivers’ from both online formats (*n* = 20) answers could be analyzed and assigned to 4 core themes with 12 subthemes, see also Table 4.

**Table 4.** Core themes and respective subthemes from participating informal caregivers’ evaluation of the online format

Core themes	Subthemes	Quote
**Usefulness for dealing with desire to die challenges**	Useful suggestions	“It helps to hear that this topic also concerns others and is taken seriously.”
	Shared experience	
	Increased self-confidence	
	Sensibilization for meta-communicative aspects	
**Aspects still missing from the online format**	Deepening of content	“As a carer, how can you manage to be there for the person(s) affected and offer a sympathetic ear while at the same time putting your own emotions to the side for the moment so as not to be overwhelmed by them?(…). In other words, tips in the sense of self-hygiene.”
	Self-care	
**Adequateness of online provision of the format**	Ambivalence regarding distance	“I think it’s very appropriate to discuss this sensitive topic digitally because it maintains a certain distance, which makes it easier not to get too emotional.”
	Didactic realization	
	Easy access	
**Especially positive or negative aspects**	Timeframe	“I liked the dialogue, the fact that questions could be asked in between and that the group was so small.”
	Expertise and scientific Input	
	Possibility for experience exchange	

## Discussion

When confronted with severe and life-limiting disease, patients can develop a desire to die (German Guideline Program in Oncology 2020). Although the confrontation with a patients’ desire to die is often experienced as challenging, there are some recommendations for adequate reaction such as showing respect, openness, and interest (German Guideline Program in Oncology 2020). Our own research indicates that the provision of knowledge, the fostering of self-reflection, and the building of communication skills are basic competencies for dealing with desire to die – this holds true for both professionals and laypersons (Boström et al. 2022). However, due to their special roles in regard to the patient, hospice volunteers and informal caregivers report different needs for education formats meant to deliver these competencies.

In clinical practice, hospice volunteers are increasingly confronted with desires to die: even at a time and in a jurisdiction in which assisted suicide was not allowed, 19% of hospice volunteers reported to have heard requests about it from patients they accompanied (Claxton-Oldfield and Miller 2015). Moreover, volunteers play a crucial role in engaging accompanied patients in conversations about death and dying (Rodríguez-Prat and Wilson 2024). Results from our volunteer focus group let assume that, in addition to imparting knowledge about desire to die, it is also important to make training participants reflect on their attitudes and skills – both for health professionals and voluntary workers. However, volunteers placed importance on 3 specific topics: honoring of their role without a therapeutic mission, working with and distancing themselves of health professionals and holding apart personal concerns and their volunteer work regarding desire to die. These topics go beyond what is known about volunteer end-of-life-education preferences (Brighton et al. 2017).

In the evaluation of our adapted training, hospice volunteers reported a higher number of items with significant improvements compared to multi-professional palliative care providers (Boström et al. 2025). This may be, because hospice volunteers rated themselves worse at t0 than palliative care providers did, allowing for a higher increase at t1. Despite having to complete an extensive course to become a hospice volunteer in Germany (Deutscher Hospiz- und PalliativVerband e. V. 2021), volunteers are otherwise usually older and retired laypersons with no medical or psychosocial background (Varay et al. 2023). One implication might be a higher need for legal and psychosocial knowledge as well as emotional self-protection of hospice volunteers. Therefore, one can assume a high demand for desire to die trainings in this target group. Our results provide first evidence for a good acceptance of our training. Interestingly, volunteers showed no demand for online desire to die trainings, leading to 2 evaluated face-to-face trainings. Previous research on preferences in end-of-life-education for volunteers showed similar results regardless of age, as learning on the computer was not seen as appropriate for complex topics (Brighton et al. 2017). In the same study, volunteers also expressed the wish for training in skills for communication and listening as well as other aspects of end-of-life care, which our training provides. The overall positive evaluation of our adapted training suggests that hospice volunteers may have the same needs for content and methods in a desire to die training as health professionals (Boström et al. 2025), but that they benefit from a specific form of address.

Informal (family) caregivers provide a substantial amount of support and care for their loved ones with severe and life-limiting illness while at the same time dealing with their own distress and grief (Goldberg et al. 2021). Moreover, they usually cannot fall back on prior medical or psychotherapeutic training in dealing with potential desire to die in their loved ones. Therefore, being confronted with such a desire can cause intense feelings such as guilt and anger, as our focus group participants reported. First studies suggest that prolonged dealing with suicidal ideation of a loved one might also trigger anticipatory grief in informal caregivers (Lascelles 2022). The experience of accompanying a loved one in assisted dying offers unique stressors, but can also be regarded as dignifying (Ganzini et al. 2009). As desires to die often have a relational component (such as the feeling of being a burden for others, e.g. for family members) (Gudat et al. 2019; Rodríguez-Prat et al. 2019), allowing room for these emotions and their effects on informal caregivers should be part of an education format for this target group. The emotionally relieving effect of sharing experiences with people who are in similar situations is well-documented from formats such as (online) self-help groups (Pluta 2022). This effect was noted on numerous occasions by participating informal caregivers in our format: the mere knowledge that others had similar experiences and that desire to die was its own research area was considered a relief.

The participants of our online information and exchange format greatly valued the possibility to share experiences in a safe environment, indicating the high unmet emotional needs of informal caregivers (Hashemi et al. 2018). As some emphasized their wonder that desire to die even is acknowledged as an actual phenomenon in research of whose results they can benefit, one can deduct the difficulty for informal caregivers to find resources targeted at them.

As the preference for an online provision suggests, informal caregivers might have seen our online information and exchange format as a “safe (enough) space” to share their experiences with the sensitive and personal topic of desire to die (Pevac 2022). Low-threshold access from home and at any time via online provision has already been found to be beneficial for other caregivers, as it allows a better compatibility of professional occupation, care work, and attendance of relevant education formats (Johnson et al. 2022). The high demand and overall positive feedback on content, presentation and structure of our online education format suggests our approach to be a useful basis in addressing informal caregivers needs in dealing with desire to die.

## Strengths and limitations

A major strength of our study is that by developing and piloting (online) education formats on dealing with desire to die for hospice volunteers and informal caregivers, we provide the first needs-oriented offers specifically targeting these usually under-represented groups. By building onto our evaluated and highly sought-after training curriculum, we ensure sustainability while at the same time responding flexibly to current demands.

Although focus group participants showed an extraordinary creativity in naming suggestions and wishes, we were unable to implement all of them due to shortage of time and resources. Regarding generalizability, the samples of our focus groups and (online) trainings were rather small and homogenous (e.g. mostly female and barely any participants under 50), therefore perhaps not reflecting the general attitudes and needs of informal caregivers and hospice volunteers.

## Conclusions

Patients with severe and life-limiting disease express their desires to die not only with health professionals but also with hospice volunteers or their informal caregivers. Due to their close relationship to the patient and status as laypersons, these specific groups are in special need for information and training on dealing with desire to die, to help minimize their burden. To the best of our knowledge, our developed education formats are the first target-specific education formats that acknowledge and address hospice volunteers’ and informal caregivers’ needs and challenges with this topic. Our results suggest that such target-specific trainings and online information and exchange formats based on a well-tried training curriculum are feasible and much-needed. A continuous adaptation to ongoing needs and developments is crucial in this regard.
